# Supplementary material for: Enhancement of Macrophage Function by the Antimicrobial Peptide Sublancin Protects Mice from Methicillin-Resistant Staphylococcus aureus
Source: J Immunol Res. 2019 Sep 8;2019:3979352. doi: 10.1155/2019/3979352 (PMC6754899; doi:10.1155/2019/3979352)

Table S1

| Gene | Forward Primer (5’-3’) | Reverse Primer (5’-3’) | References |
| --- | --- | --- | --- |
| *GAPDH* | ACCCCAGCAAGGACACTGAGCAAG | GGCCCCTCCTGTTATTATGGGGGT | NM_001289726 |
| *IL-1β* | GCCTTGGGCCTCAAAGGAAAGAATC | GGAAGACACAGATTCCATGGTGAAG | NM_008361 |
| *IL-6* | TGGAGTCACAGAAGGAGTGGCTAAG | TCTGACCACAGTGAGGAATGTCCAC | NM_001314054 |
| *IL-8* | TTGCCTTGACCCTGAAGCCCCC | GGCACATCAGGTACGATCCAGGC | NM_011339 |
| *TNF-α* | CCTCCCTCTCATCAGTTCTATGG | CGTGGGCTACAGGCTTGTC | NM_001278601 |
| *iNOS* | GTGCTGCCTCTGGTCTTGCAAGC | AGGGGCAGGCTGGGAATTCG | NM_001313921 |
| *COX-2* | GGGCTCAGCCAGGCAGCAAAT | GCACTGTGTTTGGGGTGGGCT | NM_001313921 |
| *MCP-1* | AGGTCCCTGTCATGCTTCTG | TCTGGACCCATTCCTTCTTG | NC_010339 |
| *B7-1* | ATGCTCACGTGTCAGAGGA | GACGGTCTGTTCAGCTAATG | NM_001359898 |
| *B7-2* | TCAGTGATCGCCAACTTCAG | TTAGGTTTCGGGTGACCTTG | NM_019388 |

Figure S1


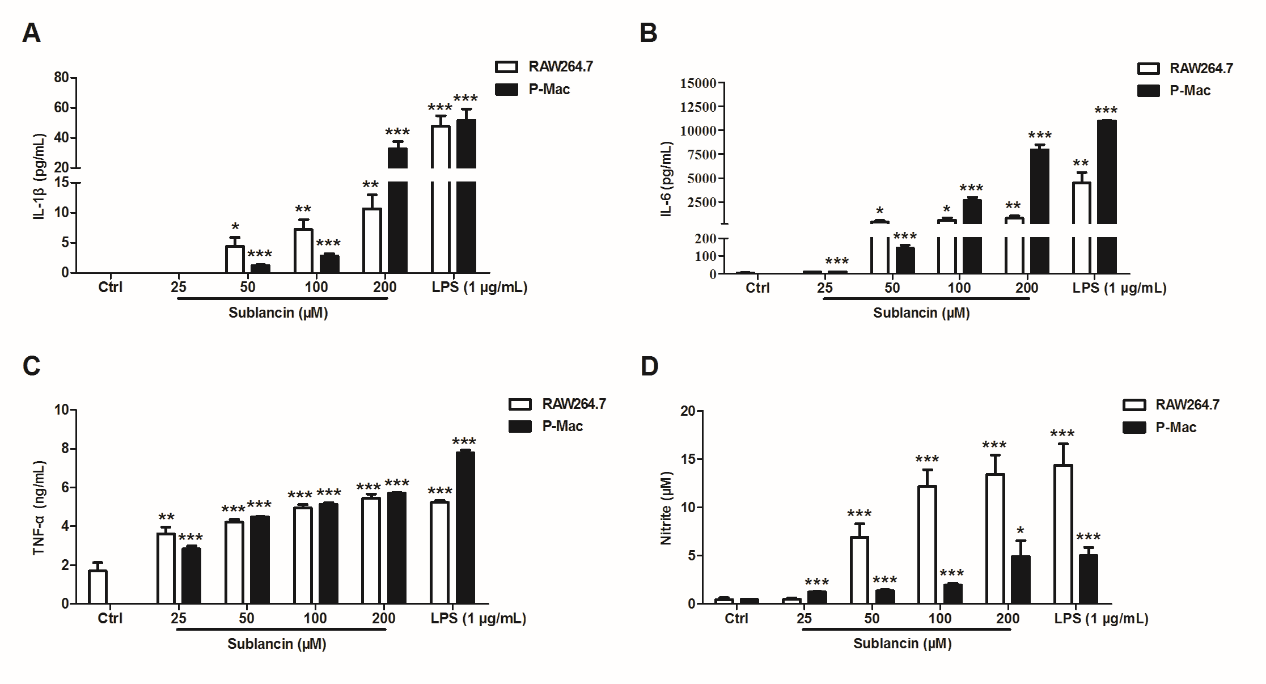


Figure S2

**
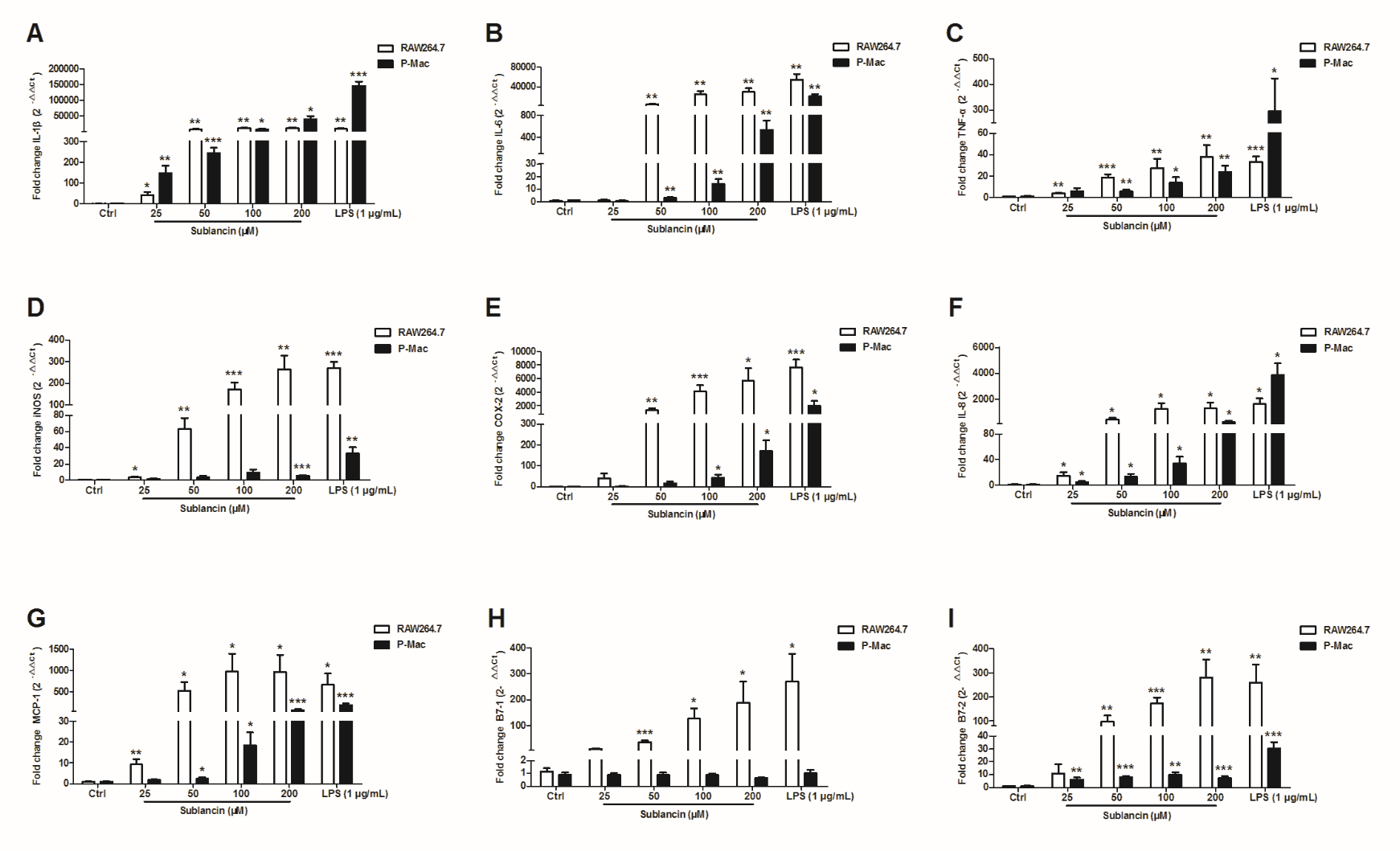
**

Figure S3

**
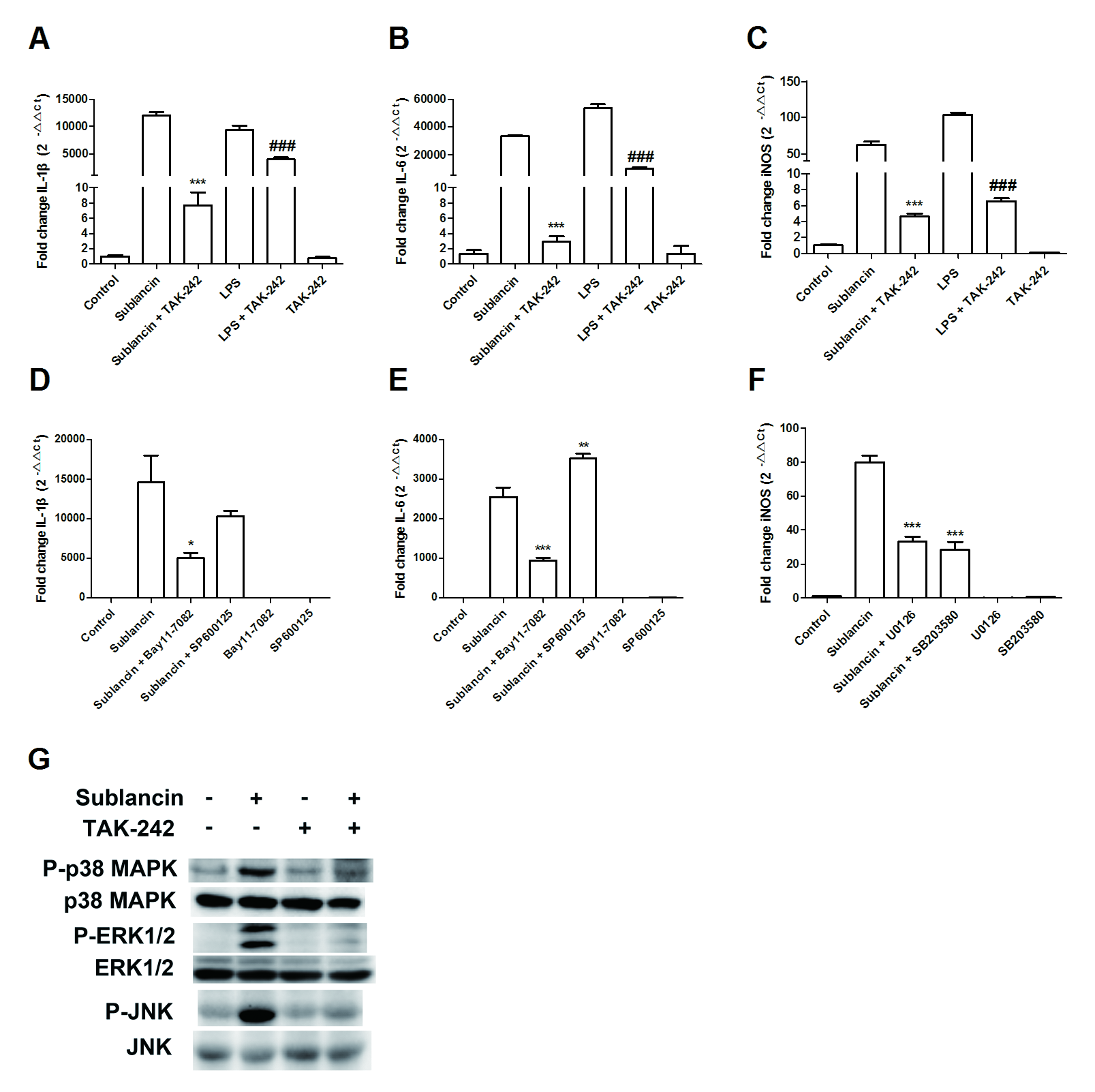
**

Figure S4


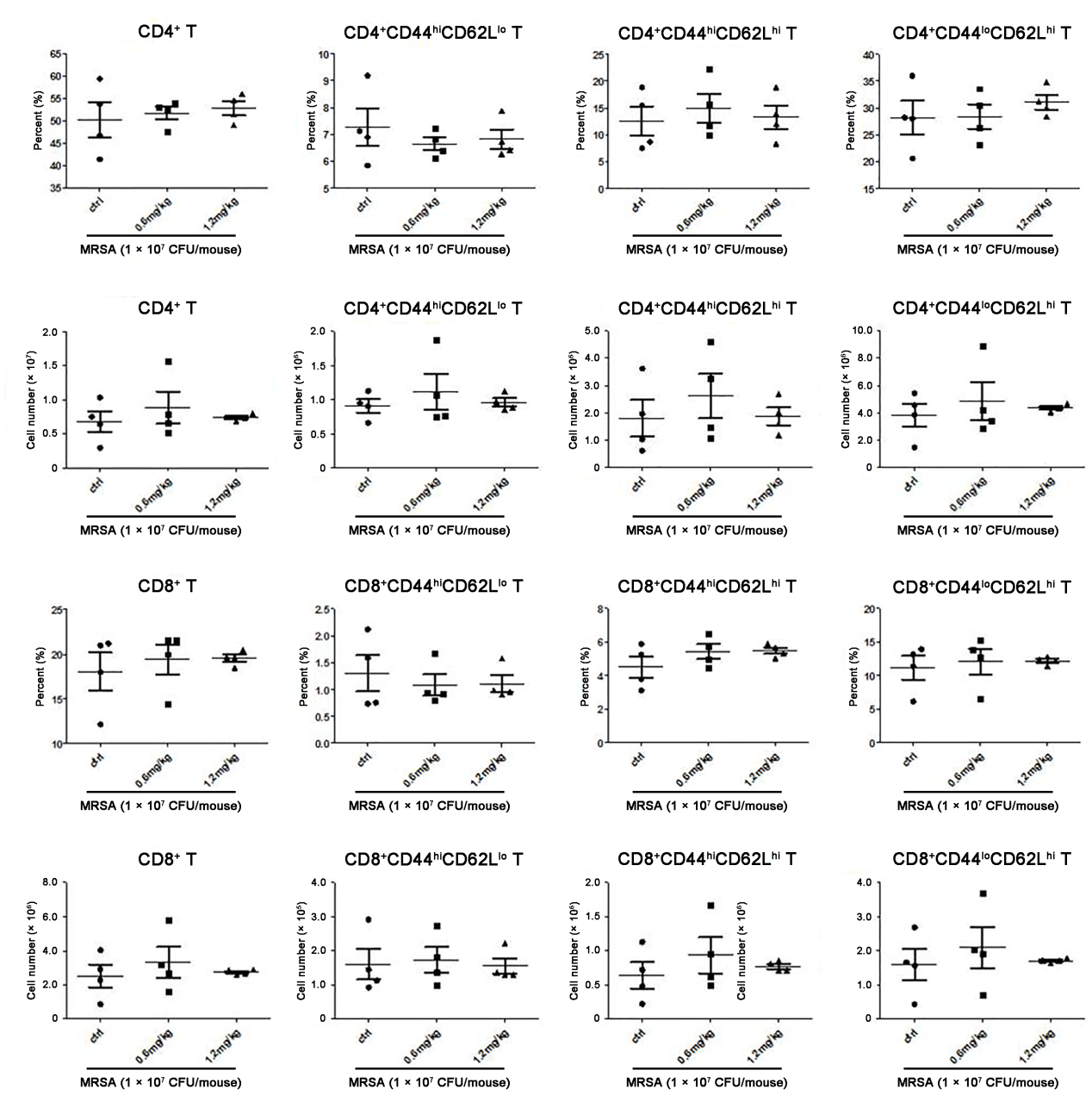

Supplement: Supplementary Materials — Table S1: sequence of the primers used for quantitative PCR. Figure S1: effect of sublancin on the production of cytokines (A to C) and nitric oxide (D) from RAW264.7 cells and mouse peritoneal macrophages (P-Mac). RAW264.7 cells and P-Mac were treated with sublancin (0-200 μM) or LPS (1 μg/ml) for 24 h. The data are expressed as mean ± SEM (n = 6). Significant differences with control cells were designated as ∗P < 0.05, ∗∗P < 0.01, or ∗∗∗P < 0.001. Figure S2: the mRNA expression of inflammatory factors (A to E), chemokines (F and G), and costimulatory molecules (H and I) in RAW264.7 cells and mouse peritoneal macrophages (P-Mac) treated with sublancin. RAW264.7 cells and P-Mac were treated with sublancin (0-200 μM) or LPS (1 μg/ml) for 12 h. The expression of target genes was detected by real-time PCR. GAPDH was used as an internal standard for normalization. The values are presented as mean ± SEM (n = 6). Significant differences with control cells were designated as ∗P < 0.05, ∗∗P < 0.01, or ∗∗∗P < 0.001. Figure S3: sublancin activated RAW264.7 cells through TLR4 signaling pathways. (A-C) Sublancin-mediated mRNA expression of cytokines via TLR4. RAW264.7 cells were pretreated for 3 h at 37°C with the TLR4 inhibitor TAK-242 (20 μg/ml) before stimulation with sublancin (100 μM) for 12 h. LPS was used as a positive control. The cell pellet was used to determine the mRNA expression of target genes by real-time PCR. Data are presented as mean ± SEM (n = 4). Compared with the sublancin-treated cells, statistical significance is shown with ∗∗∗P < 0.001. Compared with the LPS-treated cells, statistical significance is shown with ###P < 0.001. (D–F) Sublancin-mediated mRNA expression of cytokines depends on ERK1/2, p38, and NF-κB activation. RAW264.7 cells were pretreated for 30 min with the ERK1/2 inhibitor U0126 (10 μM) or the p38 inhibitor SB203580 (20 μM) or pretreated for 1 h with the NF-κB inhibitor Bay11-7082 (5 μM) or the JNK inhibitor SP600125 (20 μM) and then sti [file 3979352.f1.docx]
